# Impact of HER2 Status Assessed by Immunohistochemistry on Treatment Response in Patients with Metastatic Breast Cancer Receiving Trastuzumab Emtansine

**DOI:** 10.3390/medicina61050819

**Published:** 2025-04-29

**Authors:** Sila Oksuz, Oguzcan Kinikoglu, Ugur Ozkerim, Yunus Emre Altintas, Deniz Isik, Heves Surmeli, Hatice Odabas, Seval Ay, Tugba Basoglu, Nedim Turan

**Affiliations:** Department of Medical Oncology, Health Science University, Kartal Dr. Lütfi Kirdar City Hospital, Istanbul 34865, Turkey; ogokinikoglu@yahoo.com (O.K.); ugur.ozkerim@hotmail.com (U.O.); yunusaltintas1688@gmail.com (Y.E.A.); dnz.1984@yahoo.com (D.I.); hevessurmeli@hotmail.com (H.S.); odabashatice@yahoo.com (H.O.); drsevalay@gmail.com (S.A.); basoglutugba@gmail.com (T.B.); turan.nedim@hotmail.com (N.T.)

**Keywords:** HER2-positive breast cancer, trastuzumab emtansine, T-DM1, immunohistochemistry, progression-free survival, overall survival, resistance mechanisms

## Abstract

*Background and Objectives*: HER2-positive breast cancer accounts for approximately 20–30% of all breast cancer cases and is associated with aggressive tumor behavior. Trastuzumab emtansine (T-DM1), an antibody-drug conjugate targeting HER2, is a standard second-line therapy for patients with metastatic disease. However, the impact of HER2 immunohistochemistry (IHC) expression levels on T-DM1 efficacy remains unclear. *Materials and Methods*: This retrospective study examined 87 patients with HER2-positive metastatic breast cancer who received T-DM1 following trastuzumab-based therapy. Patients were divided into IHC 2+ and IHC 3+ groups. Progression-free survival (PFS) and overall survival (OS) were evaluated via Kaplan–Meier analysis, and group comparisons were conducted using the log-rank test. *Results*: The median progression-free survival (PFS) for the entire cohort was 7.3 months (95% CI: 5.277–9.323), with a numerically longer PFS in the IHC 3+ group (8.4 months, 95% CI: 5.915–10.952) compared to the IHC 2+ group (6.3 months, 95% CI: 4.178–8.422). However, this difference was insignificant (HR: 0.91, 95% CI: 0.61–1.35; *p* = 0.778). Similarly, the median overall survival (OS) was 23.3 months (95% CI: 18.039–28.495), with the IHC 3+ group exhibiting a slightly longer OS (24.5 months, 95% CI: 18.600–30.400) compared to the IHC 2+ group (23.2 months, 95% CI: 12.387–34.147). Again, this difference did not reach statistical significance (HR: 0.93, 95% CI: 0.63–1.42; *p* = 0.369). *Conclusions*: Although the association between HER2 IHC 3+ expression and longer PFS and OS is promising, the lack of statistical significance suggests that IHC-based HER2 stratification alone may not be sufficient to predict the response to T-DM1. The potential of conducting prospective studies with larger cohorts and comprehensive molecular profiling to refine predictive biomarkers for optimizing therapeutic outcomes in HER2-positive metastatic breast cancer is a beacon of hope and should be pursued with optimism.

## 1. Introduction

Breast cancer is one of the most common cancers affecting women today, making it a significant public health concern. This disease includes various molecular subtypes that guide treatment strategies [1]. One notable subtype is the human epidermal growth factor receptor 2 (HER2), a proto-oncogene located on chromosome 17q12-21. HER2 is overexpressed in approximately 20–30% of breast cancer cases due to chromosomal amplification, underscoring its importance in targeted therapies. The role of HER2 in signal transduction pathways is crucial as it regulates cell proliferation, survival, and differentiation, ultimately influencing tumor development and progression. HER2 forms a complex with other receptors in these pathways, activating downstream signaling cascades that promote cell growth and survival. While HER2 overexpression has traditionally been linked to a poor prognosis, it has, paradoxically, become a predictive biomarker for responsiveness to anti-HER2 therapies [2,3].

Trastuzumab, a humanized monoclonal antibody targeting the extracellular domain of HER2, has revolutionized the treatment landscape for HER2-positive breast cancer, significantly improving patient outcomes in both early-stage and metastatic settings [1,4]. Nevertheless, some patients experience primary or acquired resistance over time despite its clinical effectiveness. This underscores the importance of identifying predictive biomarkers to enhance treatment selection and facilitate the development of new therapeutic strategies. Introducing HER2-targeted therapies has considerably elevated survival rates for patients with HER2-positive diseases [5,6].

Accurate assessment of HER2 status is critical for guiding therapeutic decisions in breast cancer. Immunohistochemistry (IHC) and fluorescence in situ hybridization (FISH) are the two most commonly employed methods for evaluating HER2 expression in tumor specimens. While IHC assesses HER2 protein expression, FISH detects HER2 gene amplification [7]. In clinical practice, HER2 positivity is typically defined as an IHC score of 3+ or gene amplification confirmed by FISH, which serves as a prerequisite for treatment with HER2-targeted agents, such as trastuzumab and trastuzumab emtansine (T-DM1). However, the relationship between HER2 expression levels, as determined by IHC, and response to HER2-directed therapies, particularly T-DM1, is not always straightforward.

A growing body of evidence suggests that discordance between HER2 status in primary tumors and their metastatic counterparts may complicate treatment decisions. Although HER2 overexpression is generally associated with sensitivity to HER2-targeted therapies, emerging data indicate that even low levels of HER2 expression may influence treatment response. Retrospective studies have suggested a correlation between lower HER2 expression levels (IHC 0 or 1+) and diminished responses to trastuzumab compared with patients with higher HER2 expression [8,9]. This evolving treatment response emphasizes the need to explore the intricate relationship between HER2 expression levels and the efficacy of HER2-targeted therapies. Such investigations are essential for optimizing treatment strategies and improving patient outcomes.

The introduction of T-DM1, an antibody-drug conjugate that combines trastuzumab with the cytotoxic agent DM1, has significantly transformed the treatment landscape for HER2-positive metastatic breast cancer. T-DM1 has demonstrated greater efficacy than lapatinib and capecitabine in patients who have progressed after trastuzumab-based therapies [10]. Additionally, it can potentially address specific mechanisms of resistance to trastuzumab. This promising development allows for the targeted delivery of a cytotoxic payload specifically to cells that overexpress HER2, instilling hope and confidence for the future of HER2-positive breast cancer treatment. Moreover, evidence suggests that continuing trastuzumab treatment beyond disease progression may extend survival in patients with trastuzumab-resistant HER2-positive breast cancer [11,12].

Even with these progressions, it is essential to acknowledge that about 23% of patients with early-stage HER2-positive disease continue to experience recurrence after trastuzumab-based therapy. This highlights the critical need for predictive biomarkers to effectively stratify patients and pinpoint those most likely to benefit from HER2-directed treatment [4,13]. The influence of HER2 expression levels, assessed through immunohistochemistry (IHC), on the response to T-DM1 remains an area of ongoing research. Despite T-DM1 being approved for patients with HER2-positive metastatic breast cancer, the precise HER2 expression threshold for predicting treatment response has yet to be determined. This emphasizes the critical need for further investigation to clarify the role of HER2 expression heterogeneity in T-DM1 efficacy and to refine patient selection criteria for optimal therapeutic outcomes [11,14].

This study examined the influence of HER2 immunohistochemistry (IHC) scores on survival outcomes in patients with metastatic breast cancer who were treated with trastuzumab emtansine (T-DM1) as a second-line therapy. The results could significantly impact the HER2-positive metastatic breast cancer field, offering valuable insights that may inform future research and treatment strategies.

## 2. Materials and Methods

This retrospective analysis focused on a distinct group of patients diagnosed with HER2-positive metastatic breast cancer who received second-line treatment with T-DM1 at our institution between January 2018 and December 2024. The eligibility criteria for patient inclusion in the study were based on histologically confirmed metastatic breast cancer, HER2-positive status determined by immunohistochemistry (IHC) or fluorescence in situ hybridization (FISH), prior treatment with trastuzumab, and the availability of HER2 IHC data from metastatic lesions.

Patient demographics, tumor characteristics, prior systemic treatments, HER2 IHC scores, treatment response, progression-free survival (PFS), and overall survival (OS) were extracted from the electronic medical records. HER2 IHC status was assessed according to the American Society of Clinical Oncology/College of American Pathologists (ASCO/CAP) guidelines.

The relationship between HER2 immunohistochemistry (IHC) scores (2+ and 3+) and the treatment response to trastuzumab emtansine (T-DM1) was assessed using logistic regression analysis. The Kaplan–Meier method was employed to calculate progression-free survival (PFS) and overall survival (OS). To compare the different groups, log-rank tests and chi-square tests were utilized. Categorical variables, such as age, sex, and Eastern Cooperative Oncology Group (ECOG) performance status, were compared using chi-square and Fisher’s exact tests. The initial analysis of the relationship between clinicopathological parameters was conducted using univariate logistic regression. A *p*-value of less than 0.05 was considered statistically significant.

PFS was defined as the time from T-DM1 treatment to the date of documented disease progression. OS was defined as the duration from the initiation of T-DM1 treatment to death from any cause. Patients who did not experience the respective event by the time of the final analysis were censored at their last follow-up date. Disease control rate (DCR) refers to the percentage of patients who achieve a complete response (CR), partial response (PR), or stable disease (SD) after treatment.

All statistical analyses were conducted using IBM SPSS Statistics (version 25.0; IBM Corp., Armonk, NY, USA).

This study was conducted according to the principles of the Declaration of Helsinki and was approved by the Ethics/Institutional Review Board of Kartal Dr. Lütfi Kırdar City Hospital (date: 24 January 2025, no: 2025/010.99/12/12).

## 3. Results

A total of 87 patients with HER2-positive metastatic breast cancer treated with trastuzumab emtansine were included in this study. The median age was 48.50 (range: 25–83) years. All patients received a taxane- and trastuzumab-containing regimen as first-line treatment, followed by T-DM1 as second-line therapy.

A total of 87 patients were included, all of whom were female. Among these, 45 (51.1%) were premenopausal. At the time of diagnosis, 71 and 16 patients were classified as having ECOG 0 and ECOG 1, respectively. In addition, 58 patients (65.9%) presented with de novo metastatic disease. Of these patients, 64 (72.7%) had previously received pertuzumab in either neoadjuvant or metastatic disease settings. Regarding estrogen receptor (ER) status, 34 patients had ER < 1% (negative), two patients had ER levels between 1 and 10%, and 51 patients had ER > 10%. Among the patients, 45 (51.7%) were premenopausal and 42 (48.3%) were postmenopausal. In the premenopausal group, 12 patients showed IHC 2+ expression, whereas 33 showed IHC 3+ expression. Similarly, 12 patients had IHC 2+ expression in the postmenopausal group, and 30 had IHC 3+ expression. A total of 24 patients (27.3%) received neoadjuvant pertuzumab therapy. Visceral metastases were observed in 53 patients (60.9%) (Table 1).

**Figure 1:**
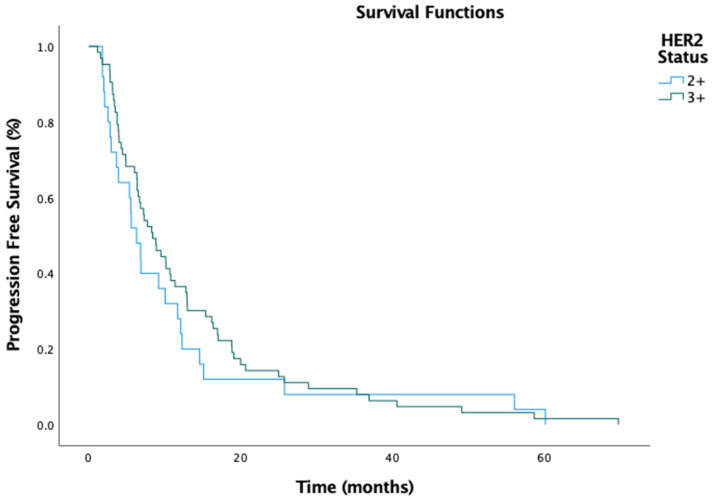
Progression-free survival by HER2 status.

**Figure 2:**
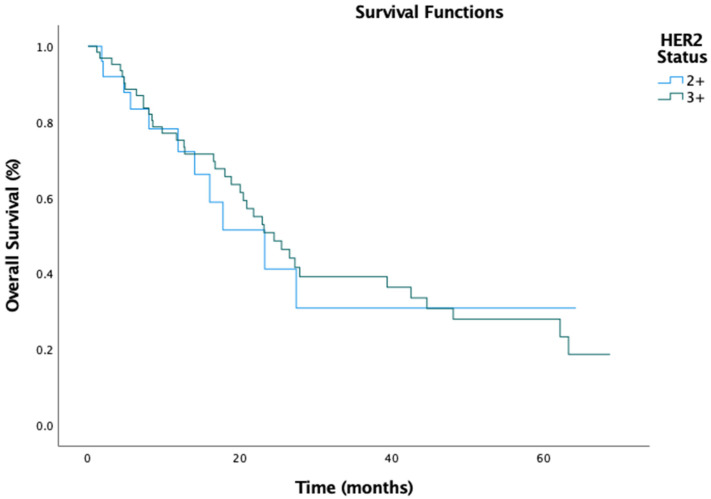
Overall survival by HER2 status.

The median PFS for the entire cohort was 7.3 months (95% CI: 5.277–9.323), with a numerically longer PFS in the IHC 3+ group (8.4 months, 95% CI: 5.915–10.952) compared to the IHC 2+ group (6.3 months, 95% CI: 4.178–8.422) (Figure 1). However, this difference was insignificant (HR, 0.91; 95% CI: 0.61–1.35; *p* = 0.778). Similarly, the median OS was 23.3 months (95% CI: 18.039–28.495), with the IHC 3+ group exhibiting a slightly longer OS (24.5 months, 95% CI: 18.600–30.400) compared to the IHC 2+ group (23.2 months, 95% CI: 12.387–34.147) (Figure 2). Again, this difference was not statistically significant (HR, 0.93; 95% CI: 0.63–1.42; *p* = 0.369).

The patients’ ECOG performance status (*p* = 0.33), menopausal status (*p* = 0.50), prior neoadjuvant pertuzumab use (*p* = 0.33), presence of visceral metastases (*p* = 0.35), and estrogen receptor (ER) positivity (*p* = 0.16) were not significantly related to progression-free survival or overall survival.

**Figure 3:**
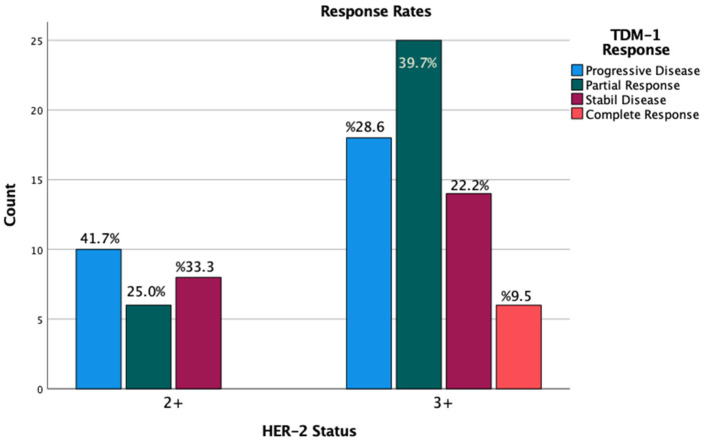
Response rates by IHC scores.

In the study cohort, the disease control rate (DCR) was 58.3% among patients with an IHC score of 2+, whereas it was 71.4% in those with an IHC score of 3+. However, this difference was not statistically significant (Figure 3).

In terms of toxicity, impaired liver function tests were observed in three patients, thrombocytopenia in four patients, and reduced ejection fraction in two patients. Treatment was discontinued in the two patients with reduced ejection fraction and in one patient who experienced grade 4 thrombocytopenia.

## 4. Discussion

HER2 expression levels are recognized to correlate with responses to HER2-targeted therapies in breast cancer [14]. In our study, the median overall survival across all patients aligned with findings from the original research [10]. However, our results revealed no significant difference in progression-free survival (PFS) and overall survival (OS) between patients with IHC scores of +2 and +3. This indicates that, within the framework of our study, HER2 expression determined by immunohistochemistry may not be a dependable predictor of treatment outcomes with trastuzumab emtansine, particularly in terms of PFS and OS.

Several factors may have contributed to these findings. First, the retrospective design of the study was inherently susceptible to selection bias, which could have influenced both the patient cohort and the treatment choices, thereby affecting the results. Second, the limited sample size may have restricted the statistical power to identify significant differences between the two groups. As a result, our study’s limited number of patients may have contributed to the lack of statistically significant findings, which should be considered when interpreting the results. Our findings align with a few other studies in this area, indicating that smaller cohort sizes lead to less reliable conclusions about the predictive value of HER2 expression in TDM1 treatment [15,16]. Another factor to consider is that patients who were initially diagnosed at an early or locally advanced stage and subsequently underwent surgery, along with the treatments they received in this setting, may have influenced our results [17].

Furthermore, other potential prognostic factors, including ECOG performance status (*p* = 0.33), menopausal status (*p* = 0.50), prior neoadjuvant pertuzumab use (*p* = 0.33), the presence of visceral metastases (*p* = 0.35), and estrogen receptor (ER) positivity (*p* = 0.16), were not found to influence either PFS or OS in this cohort significantly. These findings highlight the complexity of the treatment response in HER2-positive metastatic breast cancer and suggest that additional molecular and clinical factors may play a more critical role in determining outcomes with trastuzumab emtansine.

More extensive prospective studies are needed to investigate the role of HER2 expression in predicting response to trastuzumab emtansine. These studies aim to validate the current findings and explore whether other biomarkers or factors can more accurately predict therapeutic outcomes. Furthermore, investigating the potential of integrating HER2 expression with different molecular markers, such as PIK3CA mutations [18,19,20], EGFR and HER3 overexpression, and reprogramming mechanisms, including the mitogen-activated protein kinase (MAPK) and PI3K/AKT/mTOR signaling cascades [21,22], which have all been implicated in resistance to anti-HER2 therapy, may offer a more comprehensive understanding of the treatment response in this patient population.

This study had several limitations. First, the retrospective design might have been subject to selection bias. Second, the small sample size could have limited the statistical power to detect significant differences.

## 5. Conclusions

HER2 expression levels, determined through immunohistochemistry, may be linked to the treatment response to trastuzumab emtansine in patients with metastatic breast cancer. Nonetheless, additional prospective studies with larger sample sizes are warranted to validate these findings and establish the optimal HER2 expression threshold for predicting responses to trastuzumab emtansine.

## Figures and Tables

**Table 1:** Patients’ characteristics.

Characteristic	Number of Patients	(%)
Age		
Mean ± SD	48.50 ± 10	
Range	25–83	
ECOG PS		
0	71	80.7
1	16	19.3
ER status		
<%1	34	39.1
1–10	2	2.3
>%10	51	58.6
HER2 status		
IHC 2+	25	28.4
IHC 3+	62	71.6
Visceral metastasis		
Yes	53	60.2
No	34	28.8
Menopausal status		
Pre	45	51.7
Post	42	48.3
De novo metastasis		
Yes	57	65.9
No	30	34.1
Neoadjuvant pertuzumab		
Yes	24	27.3
No	63	72.7

Abbreviations: ECOG PS, Eastern Cooperative Oncology Group performance status; ER, estrogen receptor; HER2, human epidermal growth factor receptor 2.

## Data Availability

The data presented in this study are available upon request from the corresponding author.
